# Mechanisms and consequences of weight gain after deep brain stimulation of the subthalamic nucleus in patients with Parkinson’s disease

**DOI:** 10.1038/s41598-023-40316-0

**Published:** 2023-08-30

**Authors:** Julia Steinhardt, Laura Lokowandt, Dirk Rasche, Andreas Koch, Volker Tronnier, Thomas F. Münte, Sebastian M. Meyhöfer, Britta Wilms, Norbert Brüggemann

**Affiliations:** 1 Department of Neurology, University of Lübeck, Ratzeburger Allee 160, 23538 Lübeck, Germany; 2 Institute of Endocrinology and Diabetes, University of Lübeck, Lübeck, Germany; 3 Center of Brain, Behavior and Metabolism, University of Lübeck, Lübeck, Germany; 4 Department of Neurosurgery, University of Lübeck, Lübeck, Germany; 5 Section Maritime Medicine, Naval Medical Institute, Kiel, Germany; 6 German Center for Diabetes Research (DZD), Neuherberg, Germany

**Keywords:** Neural circuits, Neuroscience, Parkinson's disease

## Abstract

Body weight gain in combination with metabolic alterations has been observed after deep brain stimulation (DBS) of subthalamic nucleus (STN) in patients with Parkinson’s disease (PD), which potentially counteracts the positive effects of motor improvement. We aimed to identify stimulation-dependent effects on motor activities, body weight, body composition, energy metabolism, and metabolic blood parameters and to determine if these alterations are associated with the local impact of DBS on different STN parcellations. We assessed 14 PD patients who underwent STN DBS (PD-DBS) before as well as 6- and 12-months post-surgery. For control purposes, 18 PD patients under best medical treatment (PD-CON) and 25 healthy controls (H-CON) were also enrolled. Wrist actigraphy, body composition, hormones, and energy expenditure measurements were applied. Electrode placement in the STN was localized, and the local impact of STN DBS was estimated. We found that STN DBS improved motor function by ~ 40% (DBS ON, Med ON). Weight and fat mass increased by ~ 3 kg and ~ 3% in PD-DBS (all *P* ≤ 0.005). fT3 (*P* = 0.001) and insulin levels (*P* = 0.048) increased solely in PD-DBS, whereas growth hormone levels (*P* = 0.001), daily physical activity, and VO_2_ during walking were decreased in PD-DBS (all* P* ≤ 0.002). DBS of the limbic part of the STN was associated with changes in weight and body composition, sedentary activity, insulin levels (all* P* ≤ 0.040; all* r* ≥ 0.56), and inversely related to HOMA-IR (*P* = 0.033;* r* = *− *0.62). Daily physical activity is decreased after STN DBS, which can contribute to weight gain and an unfavorable metabolic profile. We recommend actigraphy devices to provide feedback on daily activities to achieve pre-defined activity goals.

## Introduction

Over the last 30 years, deep brain stimulation (DBS) of the subthalamic nucleus (STN) has become a standard method to treat therapy-resistant tremor and motor complications in advanced stages of Parkinson’s disease (PD)^1,2^. Besides its therapeutic benefits, adverse long-term side effects have been observed, including changes in the metabolic profile, energy homeostasis, endocrine signaling, and eating behavior^3^. In sum, these side effects could lead to an increase in weight together with alterations in body composition^3,4^. One predictor for postoperative weight gain appears to be the electrode position and the volume of tissue activated (VTA) within the STN. Like other basal ganglia nuclei, the STN can be divided into three functional subregions: sensorimotor, associative, and limbic subdivision, based on its connections to functionally segregated regions of the striatum, pallidum, and cortex^5,6^. Previous studies have shown that motor improvement is achieved if stimulation is done by more laterally localized electrodes in the sensorimotor area^4,7–9^, which leads to less weight gain at the same time^6,7,10–12^. In contrast, more medially located electrodes are associated with greater weight gain and a smaller reduction of motor complications. However, the association between impairments in the metabolic profile, weight gain, and the stimulation of limbic and associative STN areas is still under investigation. On the one hand, the amelioration of motor signs and the risk of lower energy expenditure due to improved motor function challenges the concept of DBS electrode localization. In this context, there is evidence that a reduction in dyskinesia scores correlates with weight gain after surgery^3,7^, indicating that improved dyskinesia symptoms could lead to weight gain at least partly due to a decrease in energy expenditure^3,7^. Alternatively, weight gain might be explained either by stimulatory effects on fiber bundles projecting from or to the hypothalamus^13,14^ or by a direct current diffusion to hypothalamic nuclei, thereby causing disruptions of regulation of endocrine signaling and perturbations in hypothalamic metabolic regulation^15–17^, such as changes in energy- and glucose-regulating hormones^3,17,18^ and alterations in energy homeostasis^19,20^.

Given the paucity of treatment recommendations to prevent weight gain and its metabolic consequences, it is important to understand the underlying pathophysiological mechanisms that drive impairments in metabolisms, such as the development of obesity or diabetes mellitus. The present study thus aims to investigate alterations in the metabolic profile, such as glucose homeostasis and, consequently, weight gain, as a side effect of STN DBS. For this, PD patients with STN DBS were measured longitudinally before and at two-time points post-surgery within one year, while PD patients under best medical treatment and healthy control subjects were included as control groups. We hypothesized that (i) PD patients with STN DBS will show increased weight and alterations in body composition compared to both control groups, while weight and body fat mass gain will be associated with stimulation in the limbic or associative subdivision of the STN; (ii) changes in relevant energy- and glucoregulatory hormones and changes in energy expenditure will occur in PD patients with STN DBS compared to control subjects and are correlated with the activation of the limbic or associative subdivision of the STN; (iii) PD patients with STN DBS will show increased daily physical activity levels which correlate with the activation of the sensorimotor subdivision of the STN.

## Results

Baseline characteristics at T_0_ are shown in Table 1. There were no dropouts in PD-DBS, whereas 17/18 patients in PD-CON (4 women) and 21/25 in H-CON (10 women) completed all time points. PD-DBS patients had higher MDS-UPDRS III and IV scores than PD-CON (*P* ≤ 0.013). BDI-II levels were higher in both PD groups compared to H-CON (*P* < 0.01), whereas MoCA revealed no significant differences at baseline (*P* = 0.118). BDI-II and MoCA remained unchanged over time in all groups (*P* ≥ 0.086). Fat mass, fasting glucose, insulin, HOMA index, growth hormone (GH), serum free triiodothyronine 3 (ft3), and resting energy expenditure (REE) were not different between groups at baseline.

**Table 1:** Demographic data and clinical variables of the study population at baseline.

Baseline (T0)	PD-DBS	PD-CON	H-CON	*P*-value
Age (years)	56.6 ± 8.4	57.9 ± 7.9	59.4 ± 8.0	0.590
Weight (kg)	81.4 ± 17.5	81.8 ± 14.0	77.3 ± 11.7	0.526
BMI (kg/m^2^)	26.7 ± 4.3	26.3 ± 4.2	25.6 ± 3.3	0.682
Waist (cm)	93.5 ± 12.5	92.4 ± 12.1	89.4 ± 12.1	0.567
Hip (cm)	105.0 ± 11.9	99.6 ± 24.6	106.0 ± 5.3	0.631
Neck (cm)	38.6 ± 5.0	39.3 ± 2.9	37.8 ± 3.6	0.401
Waist-to-hip-ratio	0.89 ± 0.08	1.38 ± 2.1	0.84 ± 0.09	0.173
Skinfold thickness (cm)	58.0 ± 22.3	56.0 ± 24.7	52.6 ± 20.5	0.741
Gender (male/female)	8/6	13/5	12/13	0.267
Age of disease onset (years)	45.7 ± 9.5	48.4 ± 7.9	N/A	0.941
Disease duration (years)	9.8 ± 4.6	9.8 ± 4.9	N/A	0.991
Handedness	0.7 ± 0.5	0.7 ± 0.5	0.9 ± 0.3	0.229
Education (years)	14.6 ± 2.9	15.9 ± 3.2	15.3 ± 2.9	0.702
MoCA	25.6 ± 1.7	28.9 ± 1.3	28.0 ± 1.6	0.118
BDI-II	7.6 ± 5.7	9.3 ± 4.9	4.2 ± 3.3^††^*	**0.003**
MDS-UPDRS-Total	56.5 ± 16.3	46.8 ± 18.4	4.5 ± 3.1	0.124
MDS-UPDRS-I	9.6 ± 5.8	10.3 ± 6.1	2.7 ± 2.5	0.948
MDS-UPDRS-II	13.4 ± 7.8	9.7 ± 6.2	0.5 ± 0.9	0.140
MDS-UPDRS-III	32.0 ± 8.1	25.2 ± 8.4^†^	1.8 ± 1.7*	**0.021**
MDS-UPDRS-IV	6.1 ± 4.6	2.1 ± 2.4^†^	N/A	**< 0.001**
Hoehn & Yahr	2.0 ± 0.3	2.0 ± 0.5	N/A	0.622
LEDD (mg/day)	833 ± 491	764 ± 512	N/A	0.838

Results are expressed as mean values ± SD. *Posthoc t-test between PD-CON and H-CON (*P* ≤ 0.003). †Posthoc t-test between PD-DBS and PD-CON (*P* ≤ 0.021). ††Posthoc t-test between PD-DBS and H-CON (*P* < 0.001). PD-DBS, patients with PD that underwent DBS surgery; PD-CON patients with PD under best medical treatment; H-CON healthy control subjects. MoCA Montreal Cognitive Assessment; MDS-UPDRS Movement Disorder Society—Unified Parkinson’s Disease Rating Scale; LEDD levodopa equivalent dose. MoCA Montreal Cognitive Assessment, BDI-II Beck’s Depression Inventory II.

Significant values are in bold.

### Clinical effects of STN DBS

GLM revealed a significant effect on MDS-UPDRS-III showing differences for the factors group (*P* < 0.001) and time (*P* ≤ 0.001), as well as a time x group interaction (*P* ≤ 0.001). Furthermore, GLM showed differences on MDS-UPDRS-IV for the main factors group (*P* < 0.001) and time (*P* = 0.027), as well as a time x group interaction (*P* ≤ 0.001). In MDS-UPDRS I and II scores, we found a significant effect of group (all *P* < 0.001), but not for the factor time (all *P* ≥ 0.243), as well as no time x group interaction (all *P* ≥ 0.386; Table 2). Next to alterations in clinical scores, we found in GLM analysis on LEDD no significant effect for the factor group (*P* = 0.264), but a significant effect for the factor time (*P* ≤ 0.001) as well as a time x group interaction (*P* = 0.016).

**Table 2:** Summary of GLM results of clinical effects of STN DBS in all groups over time.

		PD-DBS	PD-CON	H-CON	*P*-value
MDS-UPDRS I	T_0_	9.6 ± 5.8	10.2 ± 6.3*	2.7 ± 2.5^†††^	Time: *P* = 0.243 (F = 1.4)
	T_6M_	7.5 ± 5.0	8.0 ± 4.8*	2.6 ± 2.3^†††^	Group: ***P* < 0.001** (F = 26.6)
	T_12M_	9.3 ± 5.7	8.7 ± 4.1*	1.8 ± 1.9^†††^	Time × Group: *P* = 0.386 (F = 1.0)
MDS-UPDRS II	T_0_	13.4 ± 7.8	9.5 ± 6.4*	0.5 ± 0.9^†††^	Time: *P* = 0.958 (F = 0.1)
	T_6M_	10.0 ± 7.7	8.0 ± 5.9*	0.4 ± 0.9^†††^	Group: ***P* < 0.001** (F = 25.3)
	T_12M_	11.3 ± 6.6	9.1 ± 6.2*	0.2 ± 0.4^†††^	Time x Group: *P* = 0.976 (F = 0.1)
MDS-UPDRS III	T_0_	32.0 ± 8.1	25.4 ± 8.6^††^	1.8 ± 1.7^†††^*	Time: ***P* < 0.001** (F = 19.2)
	T_6M_	19.5 ± 7.3^†^	19.6 ± 9.1	1.4 ± 1.6^†††^*	Group: ***P* < 0.001** (F = 63.8)
	T_12M_	19.5 ± 20.7^†^	20.7 ± 10.9	2.3 ± 1.8^†††^*	Time × Group: ***P* < 0.001** (F = 8.2)
MDS-UPDRS IV	T_0_	6.1 ± 4.6	1.9 ± 2.2^††^	N/A	Time: ***P* = 0.027** (F = 3.8)
	T_6M_	2.8 ± 2.9^†^	2.1 ± 1.9	N/A	Group: ***P* < 0.001** (F = 17.7)
	T_12M_	2.7 ± 3.1^†^	3.1 ± 3.0	N/A	Time × Group: ***P* < 0.001** (F = 5.7)
LEDD	T_0_	833 ± 491	764 ± 512	N/A	Time: ***P* < 0.001** (F = 9.2)
	T_6M_	486 ± 406^†^	765 ± 475	N/A	Group: *P* = 0.264 (F = 1.3)
	T_12M_	428 ± 292^†^	689 ± 400	N/A	Time × Group: ***P* = 0.016** (F = 4.5)

Results are expressed as mean values ± SD. MDS-UPDRS, Movement Disorders Society Unified Parkinson’s Disease Rating Scale; LEDD, levodopa-equivalent daily dosis; N/A, not applicable; T_0_, baseline measurement; T_6M_, after 6 months of stimulation; T_12M_, after 12 months of stimulation; PD-DBS, patients with STN DBS; PD-CON, PD patients under best medical treatment; H-CON, healthy control subjects. †Posthoc t-test between baseline and T_6M_ and T_12M_ in PD-DBS (*P* ≤ 0.010). ††Posthoc t-test between PD-DBS and PD-CON (*P* ≤ 0.011). †††Posthoc t-test between PD-DBS and H-CON (*P* < 0.001). *Posthoc t-test between PD-CON and H-CON (*P* ≤ 0.001).

Significant values are in bold.

**Figure 1:**
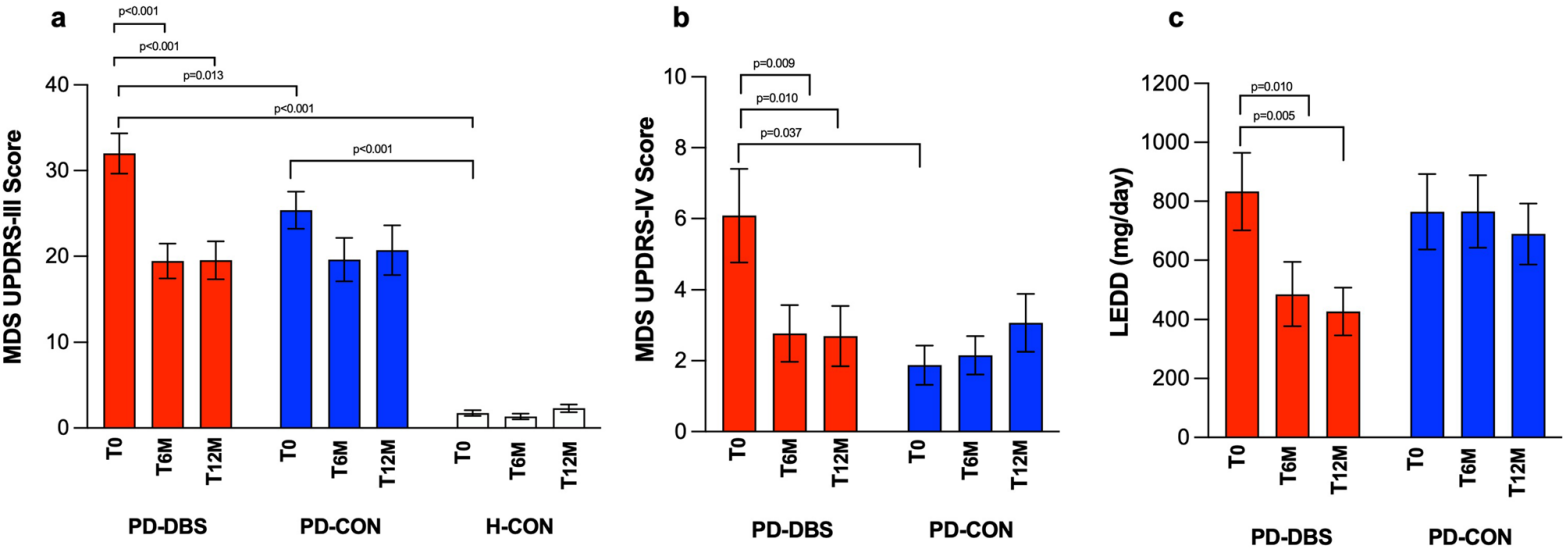
Changes in motor function (**a**), motor complications (**b**), and medication (**c**) over time. Mean change in MDS-UPDRS scores over time as a comparison between groups and time points: baseline (T_0_, first bar per group), after 6 months (T_6M_; second bar per group), and 12 months (T_12M_; third bar per group). PD-DBS, patients with STN DBS (red bars); PD-CON, PD patients under best medical treatment (blue bars); H-CON, healthy control subjects (white bars). Values are shown as mean values ± SEM.

Posthoc results showed that the MDS-UPDRS III decreased by 40 ± 2.6% at T_6M_ (*F*(2,42) = 29.3,* P* ≤ 0.001; Fig. 1a) in PD-DBS and remained stable at T_12M_ (*P* > 0.900). Differences in MDS-UPDRS-III scores were found between PD-DBS and PD-CON, PD-DBS and H-CON, as well as between PD-CON and H-CON (all* P* ≤ 0.013). MDS-UPDRS-IV scores were significantly different at baseline between PD-DBS and PD-CON (*P* = 0.037). MDS-UPDRS-IV scores decreased by 54% at T_6M_ (*P* ≤ 0.001; Fig. 1b; Table 2) and were unchanged at 2.7 ± 3.1 at T_12M_ (*P* > 0.900) in PD-DBS. LEDD decreased from 833 ± 491 mg/day at baseline by 42% to 486 ± 406 mg/day at T_6M_ (*P* = 0.010;* d* = 0.838) and remained stable at T_12M_ in PD-DBS. No change in LEDD was observed in PD-CON (Fig. 1c; Table 2).

The improvement of MDS-UPDRS-IV at T_6M_ was inversely correlated with VTA_total_ (*P* = 0.048;* r* = *− *0.61). Moreover, reduction in LEDD at T_12M_ was also inversely correlated with VTA_total_ (*P* = 0.047;* r* = *− *0.60).

### Changes in weight and body composition

GLM analysis on body weight revealed no effects for the factors group (*P* = 0.351) and time (*P* = 0.080), but a significant time x group interaction (*P* = 0.005). In line, GLM analysis on fat mass showed no effects for the factors group (*P* = 0.365) and time (*P* = 0.155), but a significant time x group interaction (*P* = 0.001).

Posthoc results revealed thereby a significant change in body weight. In PD-DBS, body weight increased by + 2.9 ± 5.0 kg (range of weight gain, − 8.2 kg to + 11.2 kg; *P* = 0.005; Table 3) at T_6M_ and + 3.2 ± 7.2 kg (− 12.6 kg to + 16.1 kg; *P* = 0.011, see Table 3) at T_12M_. In both control groups, there were no changes in body weight over time. Regarding ideal body weight, PD-DBS showed an increase of + 4.3 ± 7.0 kg (*P* = 0.004; *d* = 1.44) at T_6M_ and + 5.1 ± 10.6 kg above ideal weight (excessive weight gain (%EWG); *P* = 0.036;* d* = 1.59) at T_12M_ while there were no changes in both control groups over time. Furthermore, PD-DBS gained on average + 2.7 ± 4.1% fat mass (2.8 ± 4.8 kg; *P* = 0.002; Table 3) at T_6M_ and + 2.8 ± 5.4% (3.0 ± 6.4 kg; *P* = 0.001) at T_12M_ compared to baseline, whereas fat mass remained stable in both control groups.

**Table 3:** Summary of GLM results of body weight and body composition in all groups over time.

		PD-DBS	PD-CON	H-CON	*P*-value
Body weight (kg)	T_0_	81.4 ± 17.5	83.2 ± 13.0	77.3 ± 11.7	Time: *P* = 0.080 (F = 3.2)
	T_6M_	84.3 ± 16.7^†^	81.3 ± 11.7	76.9 ± 11.5	Group: *P* = 0.351 (F = 1.0)
	T_12M_	84.5 ± 14.7^†^	81.7 ± 12.0	77.8 ± 11.5	Time × Group: ***P* = 0.005** (F = 5.9)
Fat mass (%)	T_0_	31.8 ± 13.2	29.5 ± 10.6	34.2 ± 8.5	Time: *P* = 0.155 (F = 1.9)
	T_6M_	34.4 ± 11.6^†^	29.1 ± 11.2	33.7 ± 8.2	Group: *P* = 0.365 (F = 1.0)
	T_12M_	34.6 ± 11.1^†^	29.5 ± 10.2	34.2 ± 8.1	Time × Group: ***P* = 0.001** (F = 4.8)
Fat-free mass (%)	T_0_	68.2 ± 13.2	70.5 ± 10.6	65.8 ± 8.5	Time: *P* = 0.155 (F = 1.9)
	T_6M_	65.6 ± 11.6^†^	70.9 ± 11.2	66.3 ± 8.2	Group: *P* = 0.365 (F = 1.0)
	T_12M_	65.4 ± 11.1^†^	70.5 ± 10.2	65.8 ± 8.1	Time × Group: ***P* = 0.001** (F = 4.8)

Results are expressed as mean values ± SD. T_0_, baseline measurement; T_6M_, after 6 months of stimulation; T_12M_, after 12 months of stimulation; PD-DBS, patients with STN DBS; PD-CON, PD patients under best medical treatment; H-CON, healthy control subjects. † Posthoc t-test between baseline and T_6M_ and T_12M_ in PD-DBS (*P* ≤ 0.05).

Significant values are in bold.

VTA_limbic_ correlated with the change in weight as well as excessive weight gain at T_6M_ (*P* = 0.034,* r* = 0.57; *P* = 0.008,* r* = 0.67) and T_12M_ (*P* = 0.005,* r* = 0.70;* P* = 0.001,* r* = 0.76), and with change in fat mass at T_6M_ (*P* = 0.050;* r* = 0.53) and T_12M_ (*P* = 0.018;* r* = 0.62).

### Changes in energy expenditure

GLM revealed no significant effect on REE for the factors group (*P* = 0.072) and time (*P* = 0.174), as well as no time × group interaction (*P* = 0.101), although both groups of PD patients had slightly higher values at baseline (please see Table 4).

**Table 4:** Summary of GLM results of energy expenditure in all groups over time.

		PD-DBS	PD-CON	H-CON	*P*-value
Resting energy expenditure (kcal/day)	T_0_	1442 ± 339	1462 ± 175	1344 ± 215	Time: *P* = 0.174 (F = 1.8)
	T_6M_	1426 ± 286	1529 ± 237	1238 ± 224	Group: *P* = 0.072 (F = 2.8)
	T_12M_	1398 ± 428	1374 ± 206	1298 ± 212	Time × Group: *P* = 0.101 (F = 2.7)
VO_2_ Sitting (L/min)	T_0_	0.34 ± 0.09	0.37 ± 0.04	0.31 ± 0.05	Time: *P* = 0.527 (F = 0.63)
	T_6M_	0.37 ± 0.13	0.37 ± 0.09	0.32 ± 0.07	Group: *P* = 0.130 (F = 2.2)
	T_12M_	0.36 ± 0.09	0.35 ± 0.07	0.31 ± 0.07	Time × Group: *P* = 0.742 (F = 0.5)
VO_2_ Walking (L/min)	T_0_	1.18 ± 0.52	1.20 ± 0.26	0.97 ± 0.22	Time: ***P* = 0.030** (F = 3.7)
	T_6M_	1.19 ± 0.49	1.11 ± 0.28	1.02 ± 0.22	Group: *P* = 0.301 (F = 1.3)
	T_12M_	1.13 ± 0.45	1.09 ± 0.30	0.92 ± 0.24	Time × Group: *P* = 0.801 (F = 0.4)
Maximal heart frequency during walking (beats/min)	T_0_	160 ± 15	141 ± 31	150 ± 25	Time: ***P* < 0.001** (F = 34.8)
	T_6M_	164 ± 8	156 ± 19	138 ± 30	Group: *P* = 0.006 (F = 6.6)
	T_12M_	108 ± 15	106 ± 16	100 ± 15	Time × Group: *P* = 0.655 (F = 0.6)

Results are expressed as mean values ± SD. T_0_, baseline measurement; T_6M_, after 6 months of stimulation; T_12M_, after 12 months of stimulation; PD-DBS, patients with STN DBS; PD-CON, PD patients under best medical treatment; H-CON, healthy control subjects.

Significant values are in bold.

For VO_2_ levels during walking, GLM revealed no significant effect for the factor group (*P* = 0.301), but a significant effect for the factor time (*P* = 0.030) showing significant differences with higher values in both PD groups than in controls at baseline (*P* = 0.030; Table 4), as well as no time × group interaction (*P* = 0.801). For VO_2_ levels during sitting, GLM revealed no significant effect for the factors group (*P* = 0.130) and time (*P* = 0.527), as well as no time × group interaction (*P* = 0.742).

Concerning the heart rate during walking, GLM showed significant effects for the factors group (*P* = 0.006) and time (*P* < 0.001), but no time × group interaction (*P* = 0.655).

Additionally, rates of perceived exertion during the six-minute walking revealed a significant effect for the factor group (*P* = 0.022), but no effect for the factor time (*P* = 0.223) and no time × group interaction (*P* = 0.603) in the GLM analysis.

In PD-DBS, VO_2_ levels during walking normalized to weight decreased at T_6M_ (*P* = 0.002) and T_12M_ (*P* < 0.001) as compared to baseline, and remained stable over time in both control groups. Moreover, PD-DBS showed a higher heart rate during walking at baseline compared to both control groups (*P* = 0.013). Maximal achieved heart rate during walking decreased from 160 ± 15.4 beats/min to 108 ± 15.1 beats/min at T_12M_ (*P* < 0.001). Heart rate during walking remained unchanged over time in both control groups (Table 4). Rates of perceived exertion during the six-minute walking showed highest values in PD-DBS and lowest values in H-CON (PD-DBS: 14.1 ± 1.9, PD-CON: 11.9 ± 2.5, and H-CON: 10.5 ± 1.7; *P* = 0.002; Table 4). There was no change in perceived exertion over time within and between the groups.

### Change in daily physical activity

GLM analysis revealed for (i) total activity counts and time spent on (ii) vigorous, (iii) moderate, (iv) low, and (v) sedentary activity no effects for the factor group (all *P* ≥ 0.203), but significant effects for the factor time on (i), (ii), and (v; all P ≤ 0.023), as well as significant time × group interactions (all* P* ≤ 0.036; Table 5).

**Table 5:** Summary of GLM results of daily physical activity in all groups over time.

		PD-DBS	PD-CON	H-CON	*P*-value
Total activity counts (Counts/6 days)	T_0_	545,389 ± 287,925	330,998 ± 144,680	378,281 ± 126,615	Time: ***P* = 0.023** (F = 5.7)
	T_6M_	169,455 ± 156,247^†^	362,869 ± 151,469	408,745 ± 212,066	Group: *P* = 0.835 (F = 0.2)
	T_12M_	308,561 ± 209,066^†^	323,006 ± 127,481	456,112 ± 193,745	Time × Group: ***P* < 0.001** (F = 10.5)
Vigerous activity levels (min/ 6 days)	T_0_	227 ± 138	118 ± 56	207 ± 187	Time: ***P* < 0.001** (F = 14.1)
	T_6M_	44 ± 46	99 ± 56	135 ± 84	Group: *P* = 0.203 (F = 1.7)
	T_12M_	97 ± 87	83 ± 43	155 ± 88	Time × Group: ***P* = 0.036** (F = 3.7)
Moderate activity levels (min/6 days)	T_0_	182 ± 89	143 ± 74	133 ± 54	Time: *P* = 0.129 (F = 2.4)
	T_6M_	71 ± 64	164 ± 76	143 ± 70	Group: *P* = 0.545 (F = 0.6)
	T_12M_	119 ± 67	154 ± 64	171 ± 57	Time × Group: ***P* = 0.003** (F = 7.1)
Low activity levels (min/6 days)	T_0_	392 ± 186	300 ± 82	310 ± 111	Time: *P* = 0.106 (F = 2.4)
	T_6M_	201 ± 164^†^	357 ± 106	278 ± 102	Group: *P* = 0.923 (F = 0.1)
	T_12M_	307 ± 87	325 ± 111	356 ± 79	Time × Group: ***P* = 0.007** (F = 4.1)
Sedentary activity levels (min/6 days)	T_0_	650 ± 268	887 ± 189	791 ± 249	Time: ***P* = 0.017** (F = 6.3)
	T_6M_	1122 ± 265^†^	815 ± 195	830 ± 207	Group: *P* = 0.641 (F = 0.5)
	T_12M_	885 ± 239^†^	860 ± 197	734 ± 150	Time × Group: ***P* < 0.001** (F = 9.4)

Results are expressed as mean values ± SD. T_0_, baseline measurement; T_6M_, after 6 months of stimulation; T_12M_, after 12 months of stimulation; PD-DBS, patients with STN DBS; PD-CON, PD patients under best medical treatment; H-CON, healthy control subjects. † Posthoc t-test between baseline and T_6M_ and T_12M_ in PD-DBS (*P* ≤ 0.05).

Significant values are in bold.

**Figure 2:**
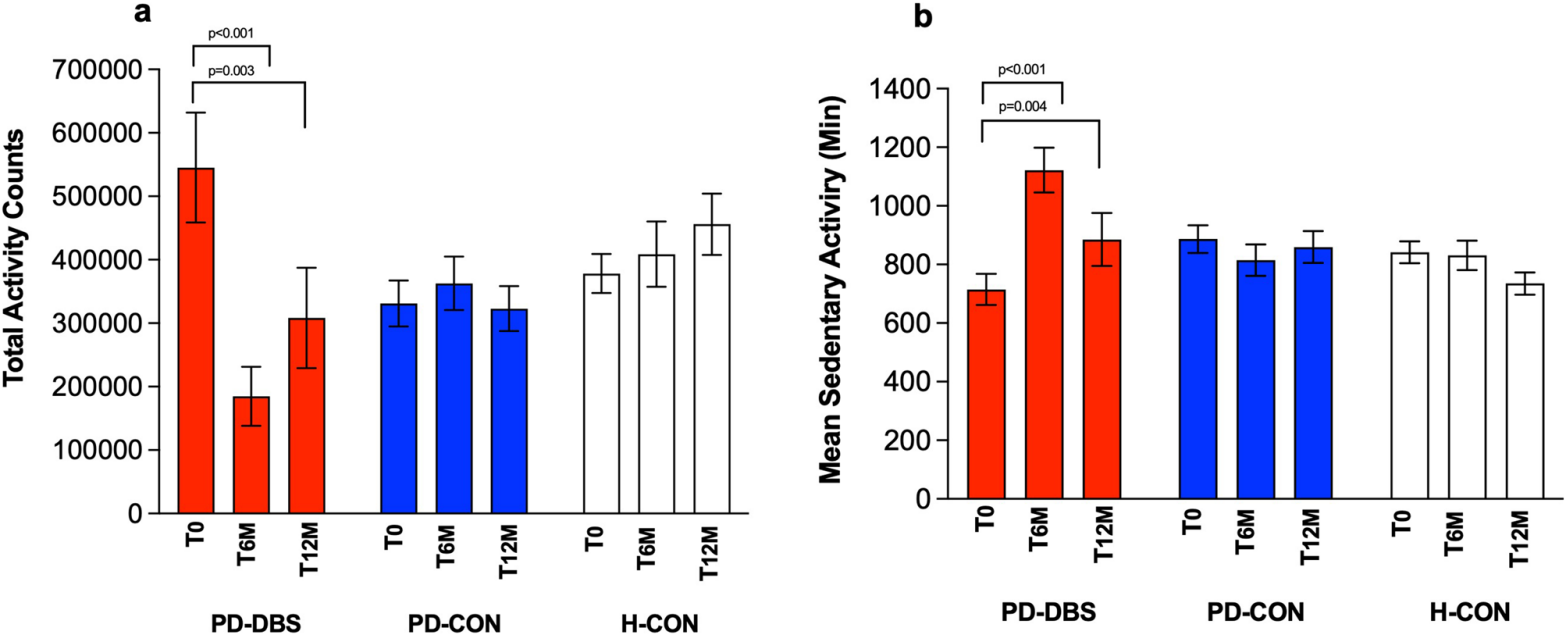
Levels in total physical activity (**a**) and sedentary activity (**b**) over time. Mean change in overall activity as well as time spent in sedentary activity over time as a comparison between groups and time points: baseline (T_0_, first bar per group), after 6 months (T_6M_; second bar per group), and after 12 months (T_12M_; third bar per group). PD-DBS, patients with STN DBS (red bars); PD-CON, PD patients under best medical treatment (blue bars); H-CON, healthy control subjects (white bars). Values are shown as mean values ± SEM.

**Figure 3:**
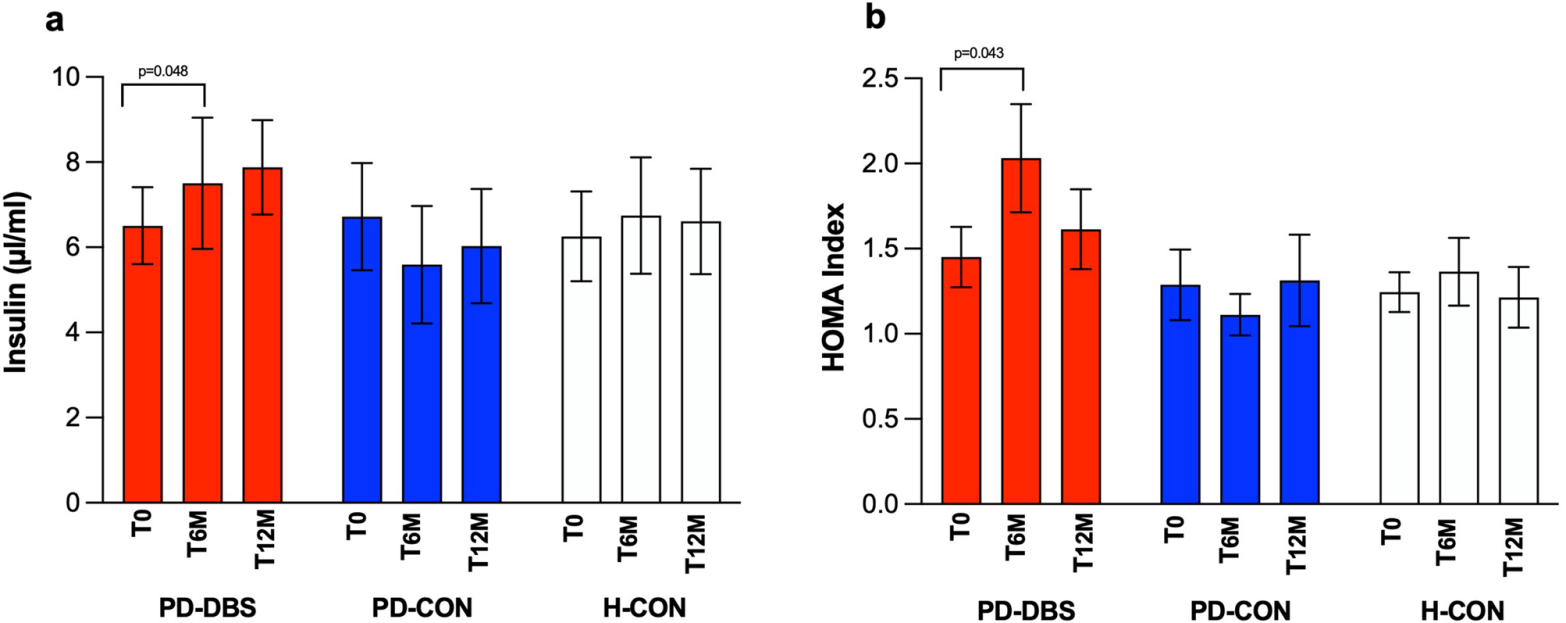
Changes in glucose homeostasis in insulin levels (**a**) and HOMA index (**b**) over time. Mean change in insulin levels and HOMA index over time as a comparison between groups and time points: baseline (T_0_, first bar per group), after 6 months (T_6M_; second bar per group), and after 12 months (T_12M_; third bar per group). PD-DBS, patients with STN DBS (red bars); PD-CON, PD patients under best medical treatment (blue bars); H-CON, healthy control subjects (white bars). Values are shown as mean values ± SEM.

In PD-DBS, total activity counts decreased by 69% from 545k ± 288k counts at baseline to 169k ± 156k (*P* < 0.001; Fig. 3a; Table 5) at T_6M_ and to 308k ± 209k (*P* = 0.003; Fig. 2a; Table 5) at T_12M_. Time spent on vigorous activity decreased from 227 ± 138 min to 44 ± 47 min (*P* = 0.036; Table 5) at T_6M_ and to 97 ± 87 min (*P* =* 0.081;* Table 5) at T_12M_. Time spent on moderate activity decreased from 182 ± 98 min to 71 ± 64 min (*P* = 0.003; Table 5) at T_6M_ and to 119 ± 67 min at T_12M_ (*P* = 0.021; Table 5). Time spent on low activity decreased from 392 ± 186 to 201 ± 164 (*P* = 0.006; Table 5) at T_6M_ and to 307 ± 87 min (*P* = 0.007; Table 5) at T_12M_, respectively. In contrast, time spent on sedentary activity increased at T_6M_ from 650 ± 268 min to 1122 ± 265 min (*P* < 0.001; Fig. 2b; Table 5) and to 885 ± 239 min (*P* = 0.004; Fig. 2b; Table 5) at T_12M_. Activity levels in all categories remained unchanged in both control groups over time.

In PD-DBS, the increased time spent in sedentary activity correlated with VTA_limbic_ (*P* = 0.012; *r* = 0.91) at T_12M_. Moreover, change in body weight and the increase in sedentary activity (*P* = 0.003; *r* = 0.506), as well as excessive weight gain with the increase in sedentary activity were positively correlated (*P* < 0.001; *r* = 0.555) at T_6M_.

At baseline, total activity counts correlated with the MDS-UPDRS IV dyskinesia item (*P* = 0.039; *r* = 0.66) in PD-DBS but neither with MDS-UPDRS III and IV total scores (Table 2), nor with the MDS-UPDRS III tremor subscore. Neither total activity counts nor changes in total activity counts at T_6M_ and T_12M_ were associated with changes in any of the scores. The same was confirmed in separate subgroup analyses for both low and sedentary activity levels.

### Change in glucoregulatory and metabolic hormones

#### Glucose homeostasis

For insulin, GLM analysis revealed no significant effects for the factor group (*P* = 0.765), but a significant effect on the factor time (*P* = 0.041), as well as no time × group interaction (*P* = 0.426). The same was true for HOMA-IR. We found no significant effects for the factor group (*P* = 0.949), but a significant effect on the factor time (*P* = 0.008), as well as no time × group interaction (*P* = 0.515).

In PD-DBS, insulin raised from 7.0 ± 2.9 µIU/ml to 9.6 ± 4.7 µIU/ml (*P* = 0.048; *d* = − 0.678; Fig. 3a; Table 6) at T_6M_. At T_12M_, insulin was 0.9 ± 1.3 µIU/ml higher compared to baseline. HOMA-IR increased from 1.5 ± 0.7 to 2.0 ± 1.1 (*P* = 0.043; *d* = 0.700; Fig. 3b; Table 6) at T_6M_ and decreased to 1.6 ± 0.9 after the increase at T_12M_. Insulin and HOMA-IR remained unchanged in both control groups over time. Fasting glucose levels remained unchanged over time in all groups.

**Table 6:** Summary of GLM results of change in insulin, growth and thyroid hormones in all groups over time.

		PD-DBS	PD-CON	H-CON	*P*-value
Insulin (µIU/ml)	T_0_	7.0 ± 2.9	7.2 ± 4.9	8.6 ± 6.9	Time: ***P* = 0.041** (F = 3.3)
	T_6M_	9.6 ± 4.7^†^	9.1 ± 9.8	8.5 ± 6.2	Group: *P* = 0.765 (F = 0.3)
	T_12M_	7.9 ± 4.1	7.6 ± 5.5	7.7 ± 5.5	Time × Group: *P* = 0.426 (F = 0.9)
HOMA-IR	T_0_	1.5 ± 0.7	1.5 ± 1.0	1.8 ± 1.5	Time: ***P* = 0.008** (F = 5.2)
	T_6M_	2.0 ± 1.0^†^	1.8 ± 1.7	1.8 ± 1.4	Group: *P* = 0.949 (F = 0.0)
	T_12M_	1.6 ± 0.9	1.5 ± 1.1	1.5 ± 1.2	Time × Group: *P* = 0.515 (F = 0.7)
Growth hormone (ng/ml)	T_0_	2.1 ± 2.0	1.1 ± 1.9	1.0 ± 1.3	Time: *P* = 0.495 (F = 0.7)
	T_6M_	0.5 ± 0.7^†^	1.8 ± 2.8	1.1 ± 1.1	Group: *P* = 0.306 (F = 2.7)
	T_12M_	1.7 ± 2.1^†^	1.7 ± 3.2	0.9 ± 1.0	Time × Group: ***P* = 0.032** (F = 2.8)
fT3 (pg/ml)	T_0_	4.0 ± 0.6	3.7 ± 0.5	3.6 ± 0.5	Time: ***P* = 0.002** (F = 6.6)
	T_6M_	3.4 ± 0.7^†^	3.6 ± 0.6	3.3 ± 0.6	Group: *P* = 0.544 (F = 0.6)
	T_12M_	3.6 ± 0.8^†^	3.7 ± 0.4	3.6 ± 0.6	Time × Group: ***P* = 0.065** (F = 2.3)

Results are expressed as mean values ± SD. T_0_, baseline measurement; T_6M_, after 6 months of stimulation; T_12M_, after 12 months of stimulation; PD-DBS, patients with STN DBS; PD-CON, PD patients under best medical treatment; H-CON, healthy control subjects. † Posthoc t-test between baseline and T_6M_ and T_12M_ in PD-DBS (*P* ≤ 0.05).

Significant values are in bold.

VTA_limbic_ was associated with changes in insulin at T_12M_ (*P* = 0.011;* r* = 0.68) and inversely correlated with HOMA-IR (*P* = 0.033;* r* = − 0.62) at T_6M_.

### Change in growth and thyroid hormones

GLM analysis revealed no significant effect on GH for the factors group (*P* = 0.306) and time (*P* = 0.495), but a significant time × group interaction (*P* = 0.032). Furthermore, GLM analysis revealed no significant effects on fT3 levels for the factor group (*P* = *0.544*), but a significant effect of the factor time (*P* = 0.002) and a trend for a significant time × group interaction (*P* = 0.065).

In PD-DBS, GH decreased from 2.1 ± 2.0 to 0.5 ± 0.7 ng/ml (*P* = 0.001; see Supplementary Fig. S1a online) at T_6M_ and increased (1.7 ± 2.1 ng/ml) at T_12M_, while levels were still lower compared to baseline (*P* = 0.050). GH levels were unchanged over time in both control groups (exact GH levels can be found in Table 6). fT3 levels lowered from 4.0 ± 0.6 pg/ml at baseline to 3.4 ± 0.7 pg/ml at T_6M_ (*P* = 0.001;* d* = 1.35) and to 3.6 ± 0.8 pg/ml at T_12M_ (*P* = 0.050;* d* = 0.574) in PD-DBS, whereas ft3 remained stable over time in both control groups (see Supplementary Fig. S1b online). There were no differences in thyroid-stimulating hormone (TSH) and fT4 over time, within and between groups.

## Discussion

In the present study, we confirm a significant weight gain as a side effect of STN DBS^7^. We could replicate the findings from a recent meta-analysis^3^ reporting an increase in weight of approximately 5 kg after one year of stimulation. In parallel, the number of patients with normal weight decreased while the number of patients with obesity class I increased by up to one-third and the observed weight gain was driven by an increase in fat mass in both, men and women. The isolated increase in fat mass was independent of gender, in contrast with the previous observation of a fat mass increase in operated women and an increase of both, fat mass and fat-free mass, in men^19–21^. Strikingly, the percentage of fat mass also increased in patients who did not gain weight or even experienced weight reduction. This argues for an unfavorable body compartment remodeling with the risk of adverse metabolic consequences^22^ such as insulin resistance and development of diabetes mellitus. However, the underlying mechanisms of this weight gain are still under discussion and likely to be multifactorial.

### Influence of DBS on the limbic system

The stimulation of the limbic subdivision of the STN was associated with alterations in weight, fat mass, glucose metabolism, physical activity, and energy expenditure. DBS could therefore influence weight homeostasis by modulating regions that impact reward, appetite, and food intake^23,24^, such as the lateral hypothalamus^25,26^, the limbic subdivision of STN^27–29^, and the medial forebrain bundle^30^. Recent FDG PET studies found a correlation between STN DBS-related weight gain and the engagement of associative and limbic brain areas, but no correlation with sensorimotor regions^4,8,9^. A recent study showed that DBS of the limbic proportion of the STN leads to increased selective attention for high-calorie foods and a sweet food-seeking-like behavior and, at the same time to a blunted response in the reward system^31^. However, if these alterations are associated with subsequent changes in dietary intake were not evaluated in respective studies. Our study also did not obtain data on food intake. Therefore, this question needs further investigation.

### Changes in motor and motivational behavior

STN DBS led to improvements in motor severity, motor fluctuations, and levodopa-induced dyskinesias in the expected range. The amelioration of motor severity, i.e., reduction in tremor, rigidity, dyskinesias, and improvement of gait, together result in reduced energy expenditure^3^. For instance, rigidity and resting tremor have been associated with a higher preoperative energy expenditure, which decreases postoperatively and leads to increased postoperative weight^3^. Another study found no change in REE, but a reduction in free-living energy expenditure^32^. In line, we found a slightly reduced REE after long-term STN DBS. However, REE is only one of three components of total energy expenditure (TEE)^17^. TEE also comprises diet-induced thermogenesis and energy expenditure (EE) related to physical activity. Previous studies in PD patients with best medical treatment revealed a reduction in TEE, which might be due to dropped activity-dependent EE^17,33^. We suppose the STN DBS may lead to a reduction in energy cost of physical activity, which then, in turn leads to lower EE. In line, movement-related EE and maximal heart frequency during walking decreased after 6 and 12 months of stimulation, both indicating less physical effort for daily activities such as walking or climbing stairs. One study measured cardiorespiratory fitness by peak oxygen uptake and showed no changes postoperatively^32^. Thus, the lower physical activity probably cannot be explained by decreased fitness. Strikingly, we found pronounced alterations in daily physical activity movement patterns after surgery. Daily levels of physical activity were reduced, and, in turn, sedentary activities were increased after surgery, also in patients who did not gain weight or even experienced weight reduction. This finding is somewhat unexpected since we assumed that patients with motor improvement will display increased levels of spontaneous physical activity. We can only speculate on the reasons for this long-term reduction in physical activity. Many non-motor symptoms have been described as a side effect of STN DBS, which could lead to reduced daily physical activity and, in consequence, body weight gain. For instance, apathy is described as a loss of motivation, decreased energy, and decreased initiative and interest^34^. Also, fatigue can be discussed in this context, although fatigue is mainly associated with weight loss during disease progression^35^. However, we found no changes in BDI-II scores after surgery over time. Furthermore, changes in apathy were most likely independent of the improvement in motor functions and reduction in dyskinesia after surgery, as shown in other studies^7,36,37^. There is also no evidence of a relationship between weight gain and improvement of motor severity and reduction of dyskinesia and motor fluctuations^19,38,39^, as well as a relationship between alterations in activity levels and changes in motor signs. This allowed us to speculate on the consequences of reduced physical activity levels and weight gain: if we assume that dietary intake remains the same while daily physical activity levels are reduced, it will result in a positive energy balance and, consequently, weight gain because of storing energy. A decrease in TEE after surgery of about 0.5 MJ/day would theoretically lead to a weight gain of about 5 kg after one year, assuming unchanged daily caloric intake postoperatively, and all extra energy was deposited as fat mass^17^.

### Co-stimulation of fibers influencing hypothalamic function

It cannot be excluded that there may be regional effects of STN DBS on hypothalamic centers depending on the exact lead position. Several hypothalamic fibers are in close proximity to the STN. Specific hypothalamic neurons are assigned to glucoregulatory properties, which could potentially be co‐stimulated due to a more medial electrode position^3^. Indeed, insulin and HOMA index were increased 6 but not 12 months after STN DBS in the present study, while fasting glucose levels remained stable over time. This hints at an incipient insulin resistance, which could accelerate the development of obesity and diabetes^40^. One possible explanation is a link between glucose metabolism and hypothalamic sensing of substrates, which can be altered after STN DBS^22^. How DBS acts on that circuitry and what exact mechanism induces insulin resistance and visceral adiposity remains elusive. However, an earlier study investigating glucose metabolism after STN DBS revealed elevated glucose oxidation rates postoperatively in DBS-treated patients^17^. One could speculate that glucose oxidation can be increased by higher insulin levels. Another study found that endogenous glucose production (EGP) was decreased during active stimulation^18^. Those changes in EGP can be influenced by insulin leading to hyperglycemia and diabetes. Therefore, STN DBS seems to affect glucose metabolism, especially if the active electrode is more located towards the limbic subdivision of the STN, thereby intervening with hypothalamic energy homeostasis.

### Strengths and limitations

An advantage of the present study is the enrollment of control groups since we can exclude that weight gain observed in patients may be linked to factors other than surgery. Due to individual differences, such as circadian influences, differences in motor impairments, or the number of meals taken, ingested calories and control for normal eating behavior should be included in future studies. Moreover, it is important to consider the limited sample size in this study which reduces statistical power, and might lead to higher variability of observed data. Furthermore, the limited sample size did not allow a subanalysis of different motor phenotypes, which could result in varying postoperative responses. Also, correlational analysis does not allow direct causation.

## Conclusions

Our findings indicate that the exact location of the active DBS contact and the modulation of the electrical field is relevant not only for the positive effects of DBS on motor symptoms but also for side effects on metabolism, like glucose homeostasis. Reduced daily activity is a possible determinant of weight gain and future studies are needed to evaluate the amount of lowered postoperative physical and its related positive energy balance. In this context, an activity tracker could be a valuable tool to provide feedback on individual activity levels. This approach could help to achieve pre-defined activity goals, e.g., the number of steps per day. Another promising option to avoid weight gain in DBS is current steering of the electrical field using segmented electrodes, allowing more fine-grained postoperative adjustments. Restricting the electrical field to the sensorimotor STN and avoiding stimulation of the limbic part of the STN may thus reduce the risk of weight gain, increase in fat mass, and predisposition to metabolic disorders such as type 2 diabetes and obesity.

## Methods

### Patients

Three groups were included: PD patients undergoing STN DBS (PD-DBS, *n* = 14 (6 females), aged 56.6 ± 8.4 years), PD patients under best medical treatment (PD-CON, *n* = 18 (5 females), aged 57.9 ± 7.9 years), and neurologically healthy control subjects (H-CON, n = 25 (13 females, aged 59.4 ± 8.0 years). The sample size was calculated based on data from a recent study comparing body mass index and body weight trajectories between PD patients treated with STN DBS compared with PD patients under best medical treatment and healthy controls before and 12 months post-surgery^3^ with an effect size of d = 2.14. We obtained sample sizes of six subjects per group (allocation: = 1.5; alpha = 0.05; power (1 − ß) = 0.95). To compensate for possible dropouts, 14 patients with Parkinson's disease that underwent STN DBS were enrolled. Since, according to our own experience, the dropout rate in the control group is higher than in groups of patients, 25 subjects have been included in the control group. PD patients were diagnosed according to the diagnostic criteria of the Movement Disorder Society^41^. PD-CON consisted of patients with comparable motor complications who would also have been eligible for DBS evaluation but who denied it. Patients with metabolic comorbidities that could affect weight (e.g., diabetes mellitus) were excluded a priori. All DBS surgeries were performed at the University Hospital Schleswig–Holstein, Campus Lübeck, by the same experienced neurosurgeons (DR, VT). The DBS-treated patients were operated on both sides, and the electrode model 3389 (Medtronic, Minneapolis, MN, USA) was implanted bilaterally. The study cohorts were matched for age, gender, weight, and BMI. All participants gave their informed written consent before the inclusion and had the opportunity to withdraw their consent at any time without a declaration of reasons. The project was approved by the Ethics Committee of the University of Lübeck (AZ17-198) and was conducted according to the Helsinki Declaration.

### Study design

The study was designed as an observational, prospective, and longitudinal cohort study over 12 months, including a baseline measurement (T_0_) as the first time point approximately two weeks before DBS surgery on stable PD medication. The second (T_6M_) and third (T_12M_) measurements were conducted 6 and 12 months, respectively, after DBS surgery (PD-DBS) or after T_0_ (PD-CON, H-CON).

The participants arrived at 8 a.m. on each examination day. They were instructed to fast overnight and drink only water or tea in the morning. All PD patients took their medication as prescribed during the measurement days (Med ON). Participants were neurologically examined by a movement disorders specialist using the Unified Parkinson’s Disease Rating Scale (MDS-UPDRS-III) and Hoehn and Yahr Scale^41^. Levodopa-equivalent daily doses (LEDD) were calculated to estimate the total individual Anti-parkinsonian drugs in milligrams of levodopa^42^.

Additionally, the Beck’s Depression Inventory was assessed (BDI-II)^43,44^. Handedness was assessed using the Edinburgh Handedness inventory^45^. Global cognitive function was tested using the Montreal Cognitive Assessment (MoCA)^46,47^. All participants were evaluated with a standard protocol which can be found as Supplementary material online. The metabolic workup comprised measurement of body weight and composition, blood sampling, measurement of energy expenditure during rest and walking. In addition, daily physical activity was assessed using wrist-accelerometry recordings (Motionwatch 8, CamNtech, Cambridge, UK). Electrodes were localized using LEAD DBS toolbox version 2.3.1 was used within MATLAB 2019 (The MathWorks, USA), and stimulation parameters^48^ (see Supplementary Table S1 online) were mapped into the standardized patient space^49^ for VTA calculation (see Supplementary Fig. S2 online). The exact procedure can be found in the Supplementary material online.

### Statistics

Data are given as mean ± SD or mean ± SEM (figures). Excel Version 2016 (Microsoft, Redmond, WA), Jamovi Version 1.8.4, and GraphPad Prism version 8.0 (La Jolla, CA) was used for analysis. One-way ANOVA was used to test for baseline differences between the metric data. Variables were checked for normality using the Kolmogorov–Smirnov test. Sphericity was tested using Mauchly’s W. In the case of non-sphericity, Greenhouse–Geisser correction was applied. The analyses of the data over time were based on a mixed general linear model (GLM), including the main factors ‘Group’ (PD-DBS vs. PD-CON vs. H-CON), and ‘Time point’ (T_0_, T_6M_, T_12M_). If GLM resulted in a significant F value with *p* ≤ 0.05 for a main effect or interaction, post hoc Student’s t-tests were performed using Bonferroni-Holm-correction. A *p*-value < 0.05 (after correction) was considered significant in all analyses. Pearson’s correlation coefficients were used for clinical data, VTA, and primary outcome parameters to test for a significant association between the two parameters. The effect size was described by Cohen’s d. In contrast, Spearman rank correlation was applied for MDS-UPDRS-III tremor and MDS-UPDRS-IV dyskinesia items. Only significant correlations are reported.

### Ethics approval

The study was approved by the Ethics Committee of the University of Lübeck (AZ17-198) and was conducted according to the Helsinki Declaration.

### Consent to participate

Informed consent was obtained from all individual participants included in the study.

### Supplementary Information

Supplementary Information.

## Data Availability

The data that support the findings of this study are available on request from the corresponding author. The data are not publicly available due to information that could compromise the privacy of research participants.
