# Supplementary material for: Mechanisms and consequences of weight gain after deep brain stimulation of the subthalamic nucleus in patients with Parkinson’s disease
Source: Sci Rep. 2023 Aug 30;13:14202. doi: 10.1038/s41598-023-40316-0 (PMC10468527; doi:10.1038/s41598-023-40316-0)
Supplement: Supplementary file 1 — Supplementary Information. [file 41598_2023_40316_MOESM1_ESM.docx]

Mechanisms and Consequences of Weight Gain After Deep Brain Stimulation of the Subthalamic Nucleus in Patients with Parkinson’s Disease – Supplementary Material

Julia Steinhardt, PhD^1,2,3^, Laura Lokowandt, MD^1,3^, Dirk Rasche, MD^4^, Andreas Koch, MD^5^, Volker Tronnier, MD^4^, Thomas F. Münte, MD^1,3^, Sebastian M. Meyhöfer, MD^2,3,6,^ Britta Wilms, PhD^2,3,5†^, and Norbert Brüggemann, MD^1,3,7†^

1 Department of Neurology, University of Lübeck, Lübeck, Germany

2 Institute of Endocrinology and Diabetes, University of Lübeck, Lübeck, Germany

3 Center of Brain, Behavior and Metabolism, University of Lübeck, Lübeck, Germany

4 Department of Neurosurgery, University of Lübeck, Lübeck, Germany

5 Naval Institute of Maritime Medicine, Kronshagen, Institute of Experimental Medicine, Section Maritime, Medicine Christian-Albrechts-Universität, Kiel, Germany

6 German Center for Diabetes Research (DZD), Neuherberg, Germany

7 Institute of Neurogenetics, University of Lübeck, Lübeck, Germany

**^†^These authors contributed equally to this work.**

**Correspondence to:** Norbert Brüggemann, MD, Dept. of Neurology and Institute of Neurogenetics, University of Lübeck; Ratzeburger Allee 160; 23538 Lübeck

*E-Mail*: [norbert.brueggemann@neuro.uni-luebeck.de](mailto:norbert.brueggemann@neuro.uni-luebeck.de)

# Materials and methods

*Study design*

All participants were evaluated with a standard protocol:

- Assessment of body height with a stadiometer [1,2], body weight [3,4] and composition with the Bod Pod (COSMED, Rome, Italy) [3,4]. Excessive weight gain in percentage (%EWG) was calculated using the following formula (adapted from Deitel 2007) [5] :

$$\%EWG=100 x\frac{(postoperative body weight-preoperative body weight)}{ideal weight}$$

- Standard blood samples were obtained at 9.00 a.m. under fasting condition. HOMA-IR index was used as an indicator for insulin resistance and calculated by multiplying glucose with insulin levels and divided by 405. The participants received a standardized breakfast after blood withdrawal calculated according to their energy demands.
- Daily physical activity was assessed using wrist-accelerometry recordings (Motionwatch 8, CamNtech, Cambridge, UK) for six consecutive days on the non-dominant arm. The data are gathered at 50 Hz and processed into ‘epochs’ of set periods of time, generally at 1-min intervals [6]. Total activity counts were defined as the total activity counts independent of the level of activity. The threshold values were set at 0-50 counts per minute for sedentary activity, 51-499 counts per minute for low activity levels, 500-999 counts per minute for moderate and >1000 counts per minute for vigorous activity levels. We calculated two additional scores to better interpret the actigraphy data: MDS-UPDRS tremor items (3.15, 3.16, 3.17, 3.18) and MDS-UPDRS dyskinesia item (4.1) at that body site where the Motionwatch was worn.
- Resting energy expenditure (REE) [7] was assessed using two indirect calorimetry devices (Vmax 29, CareFusion, San Diego, California, USA) with a ventilated hood system over a period of 30 minutes. VO_2_, VCO_2_, and ventilation were measured [8,9]. REE was computed as the mean value for 20 minutes after 10 minutes of acclimatization. REE was measured in kcal/day (body mass-independent) and adjusted to changes in weight and body composition.
- Energy expenditure (EE) during walking was assessed using a portable chest-worn indirect calorimeter (METAMAX 3B, Cortex Biophysics, Leipzig, Germany) to measure VO_2_, VCO_2_, and ventilation over a period of 11 minutes. The first five minutes represented a baseline measurement of energy expenditure during sitting. The participants were then asked to walk for six minutes with their gait speed during normal walking over a standardized walking area of 40 m back and forth. The time used for every 20 m was measured to ensure the same walking speed at T_6M_ and T_12M_ and guarantee the same amount of physical activity over the study period. VO_2_ consumption was normalized to fat-free mass. In addition, heart rate (Actiheart®, CamNTech, Cambridge, UK) [10] and the rate of perceived exertion using the Borg Scale were assessed.

*Electrode localization*

Pre-and postoperative MR imaging was obtained at 1.5T (Philips Achieva, Eindhoven, the Netherlands, 8-channel head coil) in PD-DBS. The LEAD DBS toolbox version 2.3.1 was used within MATLAB 2019 (The MathWorks, USA) for DBS lead visualization, modeling of the volume of tissue activated as well as localization of leads within MNI space [11]. Images were linearly coregistered to the pre-operative T1-image with the LEAD DBS toolbox using SPM12. Co-registration results were manually checked. Normalization to MNI space was performed by applying Advanced Normalization Tools (ANTs) [12] with a subsequent visual inspection, followed by a brain shift correction, because of lead-related nonlinear brain deformation [13]. The Brain shift algorithm within LEAD DBS uses a threefold linear registration and was also manually checked. The automated lead pre-localization on MR-images was performed using the TRAC/CORE algorithm [11]. Electrode trajectories were then manually adjusted to optimally fit the visible artifacts in the postoperative image if the automatic localization did not match the MR artifact.

*Modeling of the volume of tissue activated*

Stimulation parameters (Supplementary Table S1) were mapped into the standardized patient space [13]. The parameters were individually determined for each patient (selection of stimulation contacts, bipolar vs. monopolar, amplitude, pulse width, frequency), and VTA, as an approximation of the DBS-activated tissue, was modeled using the heuristic Dembek 2017 Atlas [11,13,14]. Correlations with VTA were calculated for total STN (VTA_total_) and the three subparts: limbic STN (VTA_limbic_), associative STN (VTA_associative_), sensorimotor STN (VTA_motor_). Only significant correlations are reported.

**References**

1. Madden AM, Smith S. Body composition and morphological assessment of nutritional status in adults: a review of anthropometric variables. *Journal of Human Nutrition and Dietetics*. 2016;29(1):7-25. doi:10.1111/jhn.12278

2. World Health Organization. *Waist Circumference and Waist-Hip Ratio: Report of a WHO Expert Consultation, Geneva, 8-11 December 2008*. World Health Organization; 2011.

3. Albersen M, Bonthuis M, de Roos NM, et al. Whole body composition analysis by the BodPod air-displacement plethysmography method in children with phenylketonuria shows a higher body fat percentage. *Journal of Inherited Metabolic Disease*. 2010;33(S3):283-288. doi:10.1007/s10545-010-9149-8

4. Shriver L, Dollar J, Lawless M, et al. Longitudinal Associations between Emotion Regulation and Adiposity in Late Adolescence: Indirect Effects through Eating Behaviors. *Nutrients*. 2019;11(3):517. doi:10.3390/nu11030517

5. Deitel M, Gawdat K, Melissas J. Reporting Weight Loss 2007. *OBES SURG*. 2007;17(5):565-568. doi:10.1007/s11695-007-9116-0

6. Resnick B, Boltz M, Galik E, Fix S, Zhu S. Feasibility, Reliability, and Validity of the MotionWatch 8 to Evaluate Physical Activity Among Older Adults With and Without Cognitive Impairment in Assisted Living Settings. *Journal of Aging and Physical Activity*. 2021;29(3):391-399. doi:10.1123/japa.2020-0198

7. Wilms B, Schmid SM, Ernst B, Thurnheer M, Mueller MJ, Schultes B. Poor prediction of resting energy expenditure in obese women by established equations. *Metabolism*. 2010;59(8):1181-1189. doi:10.1016/j.metabol.2009.11.011

8. Oshima T, Berger MM, De Waele E, et al. Indirect calorimetry in nutritional therapy. A position paper by the ICALIC study group. *Clinical Nutrition*. 2017;36(3):651-662. doi:10.1016/j.clnu.2016.06.010

9. Lam YY, Ravussin E. Indirect calorimetry: an indispensable tool to understand and predict obesity. *European Journal of Clinical Nutrition*. 2017;71(3):318-322. doi:10.1038/ejcn.2016.220

10. Crouter SE, Churilla JR, Bassett DR. Accuracy of the Actiheart for the assessment of energy expenditure in adults. *European Journal of Clinical Nutrition*. 2008;62(6):704-711. doi:10.1038/sj.ejcn.1602766

11. Horn A, Kühn AA. Lead-DBS: A toolbox for deep brain stimulation electrode localizations and visualizations. *NeuroImage*. 2015;107:127-135. doi:10.1016/j.neuroimage.2014.12.002

12. Avants B, Epstein C, Grossman M, Gee J. Symmetric diffeomorphic image registration with cross-correlation: Evaluating automated labeling of elderly and neurodegenerative brain. *Medical Image Analysis*. 2008;12(1):26-41. doi:10.1016/j.media.2007.06.004

13. Horn A, Reich M, Vorwerk J, et al. Connectivity Predicts deep brain stimulation outcome in Parkinson disease: DBS Outcome in PD. *Annals of Neurology*. 2017;82(1):67-78. doi:10.1002/ana.24974

14. Horn A, Li N, Dembek TA, et al. Lead-DBS v2: Towards a comprehensive pipeline for deep brain stimulation imaging. *NeuroImage*. 2019;184:293-316. doi:10.1016/j.neuroimage.2018.08.068

| **Code** | **Parameters of chronic DBS after 6 months of stimulation** | | | | | | | | | | | | **Parameters of chronic DBS after 12months of stimulation** | | | | | | | | | | |
| --- | --- | --- | --- | --- | --- | --- | --- | --- | --- | --- | --- | --- | --- | --- | --- | --- | --- | --- | --- | --- | --- | --- | --- |
|  | **Contacts** | | **Amplitude (V)** | | **Pulse width (µs)** | | **Hz** | | **Impedance (Ω)** | | **Current (mA)** | | **Contacts** | | **Amplitude (V)** | | **Pulse width (µs)** | | **Hz** | **Impedance (Ω)** | | **Current (mA)** | |
|  | **L** | **R** | **L** | **R** | **L** | **R** |  | **L** | | **R** | **L** | **R** | **L** | **R** | **L** | **R** | **L** | **R** |  | **L** | **R** | **L** | **R** |
|  |  | | | | | | | | | | | |  | | | | | | | | | | |
| Patient 1 | 0- C+ | 10- C+ | 2.1 | 2.9 | 120 | 60 | 150 | 1338 | | 1144 | 1.6 | 2.5 | 0-1+ | 9- C+ | 2.1 | 3.1 | 120 | 160 | 180 | 1340 | 1112 | 1.6 | 2.8 |
| Patient 2 | 2- 3+ | 10- C+ | 1.7 | 2.4 | 60 | 60 | 130 | 2784 | | 1346 | 0.6 | 1.8 | 2- C+ | 9- C+ | 1.4 | 2.4 | 60 | 60 | 130 | 1027 | 985 | 0.9 | 2.2 |
| Patient 3 | 2- 3+ | 9- 10+ | 3.3 | 3.8 | 60 | 90 | 130 | 1367 | | 1396 | 2.5 | 2.8 | 2- C+ | 9-C+ | 2.6 | 2.7 | 60 | 60 | 130 | 2272 | 2344 | 1.1 | 1.2 |
| Patient 4 | 1- 2+ | 9- 10+ | 1.3 | 2.0 | 60 | 60 | 130 | 3201 | | 2412 | 0.4 | 0.8 | 1-2+ | 9-10+ | 1.7 | 2.4 | 60 | 60 | 130 | 1828 | 1626 | 0.9 | 1.5 |
| Patient 5 | 1-C+ | 9-C+ | 1.3 | 1.5 | 60 | 60 | 130 | 1247 | | 1144 | 1.1 | 1.3 | 1-C+ | 9-C+ | 1.3 | 1.8 | 60 | 60 | 130 | 1299 | 1155 | 1.0 | 1.6 |
| Patient 6 | 1-2+ | 9- 11+ | 2.5 | 2.8 | 60 | 60 | 180 | 2272 | | 2344 | 1.1 | 1.2 | 1-2+ | 9-11+ | 2.9 | 3.2 | 60 | 60 | 180 | 2257 | 2909 | 1.3 | 1.1 |
| Patient 7 | 1+2- | 9- 10+ | 3.5 | 2.4 | 60 | 60 | 130 | 1960 | | 2502 | 1.8 | 1.0 | 1+2- | 9-10+ | 4.3 | 3.1 | 60 | 60 | 130 | 1985 | 2880 | 2.2 | 1.1 |
| Patient 8 | 0-1+ | 8+9- | 2.9 | 3.5 | 90 | 90 | 130 | 1969 | | 2492 | 1.5 | 2.4 | 0-1+ | 8+9- | 3.0 | 3.8 | 90 | 90 | 130 | 2028 | 1801 | 1.5 | 2.4 |
| Patient 9 | / | 10-C+ | / | 4.5 | / | 60 | 130 | / | | 1087 | / | 4.1 | / | 10- C+ | / | 4.6 | / | 150 | 130 | / | 1066 | / | 4.3 |
| Patient 10 | 1-C+ | 9-C+ | 2.2 | 2.5 | 90 | 90 | 130 | 1657 | | 1397 | 1.3 | 1.8 | 1-C+ | 9-C+ | 3.0 | 2.5 | 60 | 90 | 130 | 1405 | 1366 | 2.2 | 1.8 |
| Patient 11 | 1-2+ | 9- C+ | 3.9 | 3.1 | 60 | 60 | 180 | 777 | | 1002 | 5.0 | 3.1 | 0-1+ | 9-C+ | 4.2 | 3.6 | 60 | 60 | 180 | 750 | 840 | 5.6 | 4.3 |
| Patient 12 | 2+1- | 10- C+ | 1.9 | 2.7 | 60 | 60 | 160 | 528 | | 914 | 3.5 | 2.9 | 1+2- | 10- C+ | 2.0 | 2.8 | 60 | 60 | 130 | 570 | 867 | 3.2 | 3.1 |
| Patient 13 | 2-C+ | 10-C+ | 2.2 | 2.7 | 60 | 60 | 130 | 1121 | | 1248 | 2.0 | 2.2 | 2-C+ | 10- C+ | 2.2 | 2.7 | 60 | 60 | 130 | 1121 | 1248 | 2.2 | 2.5 |
| Patient 14 | 0-1+ | 9-10+ | 3.7 | 4.1 | 90 | 90 | 130 | 2056 | | 1280 | 1.8 | 3.2 | 0-C+ | 10- C+ | 2.0 | 2.9 | 90 | 90 | 130 | 1598 | 988 | 1.3 | 2.9 |

**Supplementary Table S1. Stimulation settings for all patients individually described by contacts, voltage, pulse width, frequency, impedance, and current.**

*Notes*. Stimulation settings for all patients individually described by contacts, voltage, pulse width, frequency, impedance, and current for chronic DBS.

**Supplementary Table S2. Length and coordinates of individual electrodes within the STN in the left and right hemisphere in MNI space.**

|  | **Right Hemisphere** | | | **Left Hemisphere** | | |
| --- | --- | --- | --- | --- | --- | --- |
|  | x | y | z | x | y | z |
| *Patient 1* | 9.86 | -15.39 | -10.60 | -11.07 | -17.38 | -8.50 |
| *Patient 2* | 7.88 | -15.39 | -10.67 | -10.82 | -17.91 | -11.17 |
| *Patient 3* | 11.03 | -15.86 | -8.98 | -11.63 | -16.72 | -8.84 |
| *Patient 4* | 10.84 | -15.62 | -9.45 | -9.42 | -16.45 | -10.91 |
| *Patient 5* | 12.55 | -14.64 | -10.51 | -11.16 | -20.14 | -12.21 |
| *Patient 6* | 9.66 | -18.55 | -9.59 | -8.48 | -16.15 | -10.15 |
| *Patient 7* | 8.72 | -19.06 | -9.62 | -6.96 | -16.89 | -6.94 |
| *Patient 8* | 11.77 | -14.84 | -7.66 | -13.81 | -15.79 | -6.63 |
| *Patient 9* | 5.76 | -13.09 | -9.33 | -6.82 | -14.11 | -8.98 |
| *Patient 10* | 10.10 | -16.53 | -6.91 | -9.64 | -16.20 | -10.29 |
| *Patient 11* | 9.69 | -12.72 | -7.42 | -10.43 | -16.56 | -7.04 |
| *Patient 12* | 11.84 | -13.67 | -8.58 | -9.43 | -14.64 | -8.38 |
| *Patient 13* | 10.89 | -14.66 | -10.23 | -9.94 | -15.26 | -10.08 |
| *Patient 14* | 8.83 | -16.06 | -7.88 | -13.84 | -15.57 | -7.66 |
| *Mean* | 9.96 | -15.43 | -9.10 | -10.25 | -16.41 | -9.13 |
| *SD* | 1.78 | 1.80 | 1.24 | 2.09 | 1.48 | 1.73 |

**Supplementary Figure S1**

**
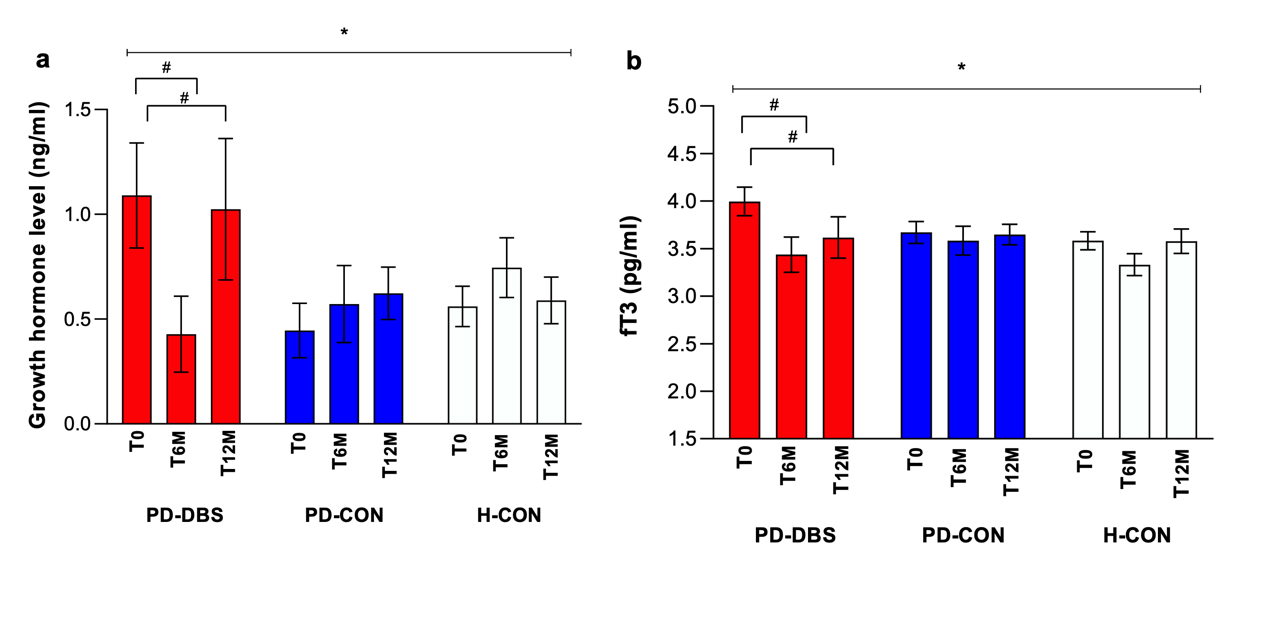
**

**Supplementary Figure S1 Changes in energy-regulating hormones**. Mean change in GH and fT3 levels over time as a comparison between groups and time points: baseline (T0, first bar per group), after 6 months (T6M; second bar per group), and after 12 months (T12M; third bar per group). PD-DBS, patients with STN DBS (red bars); PD-CON, PD patients under best medical treatment (blue bars); H-CON, healthy control subjects (white bars). (A) GH levels and (B) fT3 levels. Values are shown as mean values±SEM. *rmANOVA, p≤0.05; # Bonferroni Posthoc test, p≤0.05.

**Supplementary Figure S2**

**
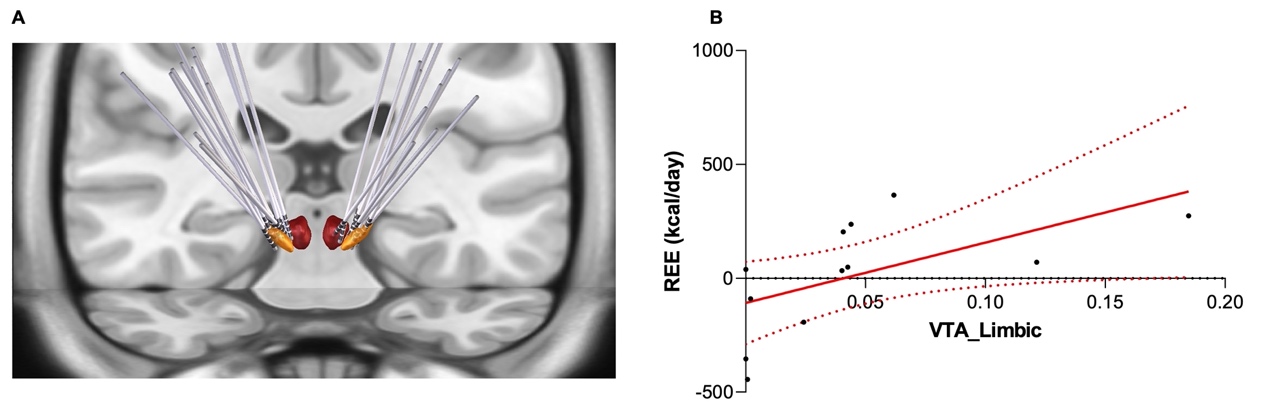
**

**Supplementary Figure S2 Target report of group electrode localization.** Electrodes are depicted in both hemispheres. The STN is shown in orange.
